# Tubal Blockage Surgery: A Retrospective Cohort Study on Clinical Characteristics and Reproductive Outcomes Within Six Years

**DOI:** 10.7759/cureus.39879

**Published:** 2023-06-02

**Authors:** Amin-Florin El-Kharoubi, Florin Szasz

**Affiliations:** 1 Obstetrics and Gynecology, University of Oradea, Oradea, ROU

**Keywords:** neosalpingostomy, diagnostic laparoscopy, tubal recanalization, tubal infertility, female fertility

## Abstract

Introduction and objectives

This research undertakes a comprehensive evaluation of demographic information and medical antecedents, in addition to intraoperative observations, for patients diagnosed with tubal obstruction. Furthermore, we delineate the therapeutic procedures implemented to achieve bilateral tubal patency. The overarching objective of this study is to ascertain the efficacy of the aforementioned therapeutic procedures and to establish an optimal timeframe before the necessity for exogenous intervention becomes apparent.

Material and methods

This study conducted a retrospective analysis of patients diagnosed with infertility due to tubal obstruction at the Oradea County Clinical Hospital, spanning a six-year period from 2017 through 2022. We evaluated numerous factors, including demographic data of the patients, intraoperative observations, and the exact site of the obstruction within the fallopian tubes. Additionally, we monitored patients post-procedure to assess their potential for fertility following the intervention. Our study involved a comprehensive examination of 360 patients in total. The primary objectives of our research were to provide clinicians with significant insights regarding the likelihood of spontaneous conception subsequent to surgical interventions and to propose guidelines on establishing an adequate waiting period prior to recommending other interventions. We employed a mix of descriptive and inferential statistical methods to analyze the data amassed.

Results

This study encompassed an initial patient population of 360 included in the study following specific exclusion criteria; the remaining 218 patients constituted the study cohort. The mean±SD age of the patients was 27.9±4.4.

Out of the entire cohort, 47 patients presented with minimal adhesions, while 117 patients exhibited blockages in one fallopian tube. A total of 54 patients were diagnosed with bilateral tubal defects. Post-intervention, patients were monitored and it was noted that 63 patients achieved pregnancy. The correlation analysis indicated the significant impact of tubal defect characteristics and patient age on fertility outcomes. The most favorable fertility outcomes were observed to be influenced by factors such as patient age and blockage location, while a higher body mass index (BMI) was found to exert a negative impact on fertility. Temporal analysis revealed that 52 patients conceived within the initial six months post-intervention, whereas only 11 patients became pregnant in the subsequent months.

Conclusions

Our research indicates that age, parity, and tubal damage severity predict tubal intervention success. Fimbriolysis was the most successful, while outcomes for salpingotomy varied. Conception significantly declined 12 months post-intervention, suggesting this is a reasonable waiting limit for a successful pregnancy.

## Introduction

Infertility is a public health issue, according to the World Health Organization. The American College of Obstetricians and Gynaecologists defines it as "failure to obtain pregnancy after 12 months or more of unprotected intercourse for women under 35 and after six months in women over 35" [1]. According to studies on couple infertility, women were the cause for around 35% of infertility in couples, and of female infertility causes that can be identified, the tubal factor affects about 20% of women [2]. Tubal infertility refers to a condition in which a woman's fallopian tubes are blocked, damaged, or otherwise unable to function properly, leading to difficulties in conceiving a child. The fallopian tubes play a crucial role in the reproductive process, as they are responsible for transporting the egg from the ovary to the uterus, and also provide the site for fertilization of the egg by the sperm. Tubal infertility can result from a number of factors, including pelvic inflammatory disease, endometriosis, previous pelvic surgery, or a history of sexually transmitted infections. In some cases, surgical intervention may be necessary to address the underlying issues with the fallopian tubes and improve a woman's chances of conceiving. The specific medical history of the individual female, as well as the variety and degree of damage to the tubal tissue, need to be taken into consideration in order to identify the type of surgery that will be performed.

Laparoscopy is still the gold standard for diagnosing infertility in women, and it is not uncommon for further therapy to be indicated after the first examination. This is done in the hopes of improving or regaining the individual's fertility in order to provide the quickest favorable outcomes based on a person's characteristics. Therefore, studies on the prospects of specific surgical treatments on the fallopian tubes and their effects on fertility must be regularly updated.

This retrospective cohort study evaluates 218 patients under the age of 35 who correspond to the definition of infertility and in whom, following examinations for the cause of infertility, tubal infertility was established. In order to determine which procedures are the best for different situations, data on the patient's medical history, paraclinical parameters, description of intraoperative observations, and evaluation of fertility results in the following year without interfering in any way with reproduction were evaluated and analyzed.

## Materials and methods

This retrospective cohort study took place at Spitalul Clinic Judetean de Urgent Oradea in Bihor, Romania. We examined the medical records of 360 patients aged 20-35 who visited our infertility clinic for treatment between 2017 and 2021. All patients were diagnosed with infertility. As part of the investigation into the cause of infertility, every patient underwent a laparoscopic procedure which determined that the fallopian tubes were the source of infertility. The study also included a thorough examination of the patients' overall medical histories and a review of all collected clinical and paraclinical information. Direct observations were made during a surgical procedure using laparoscopic techniques to assess the reproductive organs and identify the underlying causes of infertility. Subsequently, a series of laparoscopic procedures were performed to address the discovered issues and enhance the patients' chances of natural conception. The success of these surgeries was then compared to the patients' fertility in the subsequent year, without using any assisted reproduction methods. To focus on potential pathologies causing infertility, the study included only patients for whom male-related infertility causes had been eliminated through testing and evaluation. Additionally, only patients with a healthy ovarian reserve, as determined by the antimullerian hormone assessment, were included. This approach ensured that male factors or diminished ovarian reserves did not confound the study, allowing for the acquisition of valuable insights into the most effective treatments for tubal infertility in similar patients.

Ethical clearance and informed consent were obtained from the participants.

To ensure a thorough and meticulous study, stringent inclusion and exclusion criteria were enforced for the patient population. Each patient underwent pelvic laparoscopy to examine tubal patency and ascertain if a tubal factor was the primary cause of their infertility. The study's exclusion criteria were also carefully established to include only patients who met specific requirements. Patients above 35 years old were excluded, as active intervention through assisted reproductive programs is commonly recommended for them. Furthermore, patients with a history of fallopian tube interventions or those identified to have another simultaneous cause of infertility were excluded from the study. Patients with other pathologies or extrauterine pregnancies during the study period were also excluded.

Our study employed a combination of descriptive and inferential statistics to analyze the data collected. Descriptive statistics were used to summarize and present the data in a clear and concise manner, using measures such as mean and standard deviation, as well as graphical and tabular representations. These descriptive statistics provided a comprehensive overview of the data, allowing for a better understanding of the overall trends and patterns present.

Inferential statistics were then used to evaluate the relationships between different variables and test for statistical significance. To conduct this analysis, the data were analyzed by IBM SPSS software v. 25.025 (IBM Corp., Armonk, NY) and subjected to Pearson's correlation test. The results of these tests were presented using average SEM (standard error), The univariate Cox regression model was also employed, and the results were expressed as a 95% confidence interval (CI) in terms of the hazard ratio. The normal distribution of the data was assessed using the Kolmogorov-Smirnov test.

In our infertility clinic, we prioritized conducting an in-depth assessment of each patient's medical history and comprehensive clinical and paraclinical examinations. After completing these initial evaluations, patients with no other causes of infertility underwent a laparoscopy to examine the internal genital organs and assess tubal patency. During this procedure, the tubal permeability test was conducted using a uterine cannula and methylene blue to determine the presence of a tubal cause. If a treatable tubal cause was identified during the same intervention, it was promptly addressed. If one or both fallopian tubes were obstructed, the blockage's specific location dictated the type of surgical intervention required. For example, if the blockage is at the fimbrial level, fimbriolysis may be performed. In cases where the blockage occured at the infundibular level, salpingostomy might be employed. This procedure involves creating an incision in the fallopian tube and making a new opening. The incision's dimensions can vary depending on the blockage's severity, typically up to 2 centimeters. After the surgical intervention, the incision edges were cauterized to produce abrasion, which helps prevent scar tissue formation that could further block the fallopian tube. In the case of those with blockage at the ampullary level, the intervention is anastomosis after cutting the defect. Patients whose issues were successfully resolved were monitored to confirm fertility restoration.

Demographic characteristics (age), weight and body mass index, the patient's history as well as obstetric history, history of abortions and their type, history of pelvic inflammatory disease, and presence of vaginal infection were collected through interviews and examination.

All of the cases included in this study were actively attempting to conceive a child and were followed up for a period of six months to one year. During this time, the patients were closely monitored throughout the study period to assess for the presence of any crude intrauterine pregnancy (IUP). A result was considered to be positive if an intrauterine pregnancy was confirmed via ultrasound; patients whose pregnancy implanted outside the uterine cavity were excluded from the study according to the established exclusion criteria. In total, 218 patients met the inclusion criteria. The study protocol was approved by the Local Research Ethics Committee at Oradea University of Medical Sciences, Oradea, Romania.

**Figure 1:**
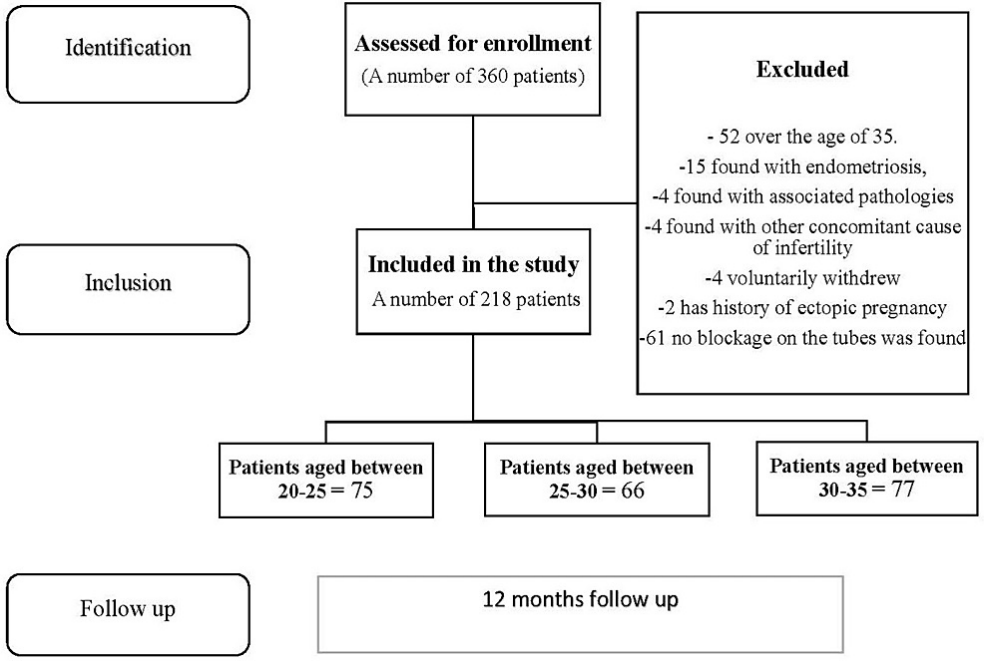
Summarization of the inclusion and exclusion data process

Figure 1 is a diagram outlining the process of patient selection.

Out of the 360 patients diagnosed with tubal infertility in our records who met the inclusion criteria, 52 were excluded from the final analysis due to being over 35 years of age. Moreover, 15 patients were excluded due to endometriosis, a condition that could significantly impact our results. Another four patients were excluded due to the development of associated pathologies during the study period (three cases of ovarian cysts requiring intervention and one case of hydrosalping). Furthermore, four patients were excluded because of another concomitant cause of infertility, which could potentially affect the results' accuracy; all four had thyroid pathology. Four patients voluntarily withdrew from the study. Lastly, two patients were excluded due to a history of ectopic pregnancy and tubal surgery, and 61 patients were excluded after performing a laparoscopy that revealed mobile tubes with no blockage. The remaining total of 218 patients had their clinical and paraclinical data, medical history, and intraoperative observations and interventions analyzed. Additionally, their fertility outcomes were observed and recorded over the next 6-12 months following the intervention. This information offers valuable insights into the intervention's effectiveness and enhances the understanding of its impact on patients' fertility. The analysis will likely contribute to the development of more effective treatments for similar conditions in the future.

## Results

The mean±SD age of the patients was 27.92±4.472 years. When presenting the clinical and paraclinical data, it was determined that grouping patients into three five-year age categories would be the most effective approach, allowing for a clearer understanding of the data and more meaningful comparisons between different age groups. The patient population consisted of 75 (34.4%) patients aged 20-25 years (group A), 66 patients (30.3%) aged 25-30 years (group B), and 77 patients (35.3%) aged 30-35 years (group C).

Table 1 features a summary of the patients' clinical data and medical history, along with the results of Pearson's chi-square test, which were correlated with post-intervention fertility outcomes.

**Table 1:** A summary of the patients' clinical data and medical history and the Pearson's chi-square test results correlating with post-intervention fertility outcomes.

			20-25 years	25-30 years	30-35 years	Total		Pearson chi-square
Age			22.65±1.52 (n=75)	28.18±1.44 (n=66)	32.81±1.15 (n=77)	27.91±4.47 (n=218)		0.00
Weight	Body Mass Index		23.97	23.72	23.68	23.79		0.011
	Patients with Obesity		5	3	4	12		0.091
Subject history								
Obstetrics history		Primary infertility	52	48	49	149		0.192
		Secondary infertility	23	18	28	69		
History of abortion		induced	6	3	5	14	27	0.41
		spontaneous	1	6	6	13		
History of pelvic inflammatory disease			6	4	8	18		0.664
Present infections with chlamydia or trichomoniasis			-	20	-	12		0.356

Table 2 provides a display of the intraoperative findings for the patients categorized by age groups.

**Table 2:** Table representing intraoperative findings in patients, categorized by age group

Age			20-25	25-30	30-35	Total
Tubal patency	Bilaterally permeable with minimal adhesions		20	16	7	47
	Unilateral salpingotomy	Total	42	33	42	117
		Fimbrial	35	20	29	21
		Ampulary	3	7	11	13
		Isthmic	4	6	2	12
	Bilateral salpingotomy	Total	9	17	28	54
		Fimbrial	9	13	23	45
		Ampulary	-	2	1	3
		Isthmic	-	2	4	6

We assessed patients' obstetric history, examining their parity status and history of abortions. We found 52 primary infertilities in Group A, 48 in Group B, and 49 in Group C. In terms of secondary infertility, there were 23 patients in Group A, 18 in Group B, and 28 in Group C.

**Figure 2:**
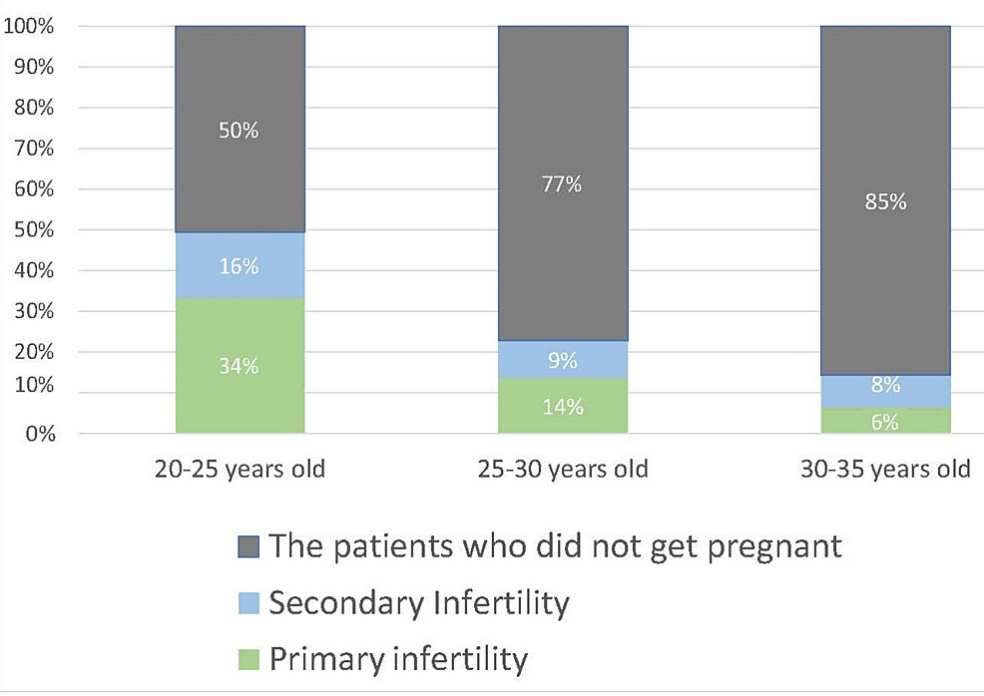
Chart illustrating, by age category, the group of patients who became pregnant, with consideration given to their obstetric history

Figure 2 illustrates, by age category, the group of patients who became pregnant, with consideration given to their obstetric history.

A total of 27 patients had experienced an abortion, with 14 induced and 13 spontaneous abortions. We identified nine patients with a previous diagnosis of pelvic inflammatory disease: six in group A, four in group B, and eight in group C. We also conducted vaginal discharge culture examinations during consultations, discovering that 20 patients in age category B had active vaginal infections caused by chlamydia or trichomoniasis. After initial assessments, laparoscopy was performed to evaluate tubal patency and conduct an exploratory examination of the pelvic cavity. Out of the entire cohort, 47 patients had minimal adhesions, while 117 patients had blockages in at least one fallopian tube. These patients were distributed among the groups as follows: 42 in Group A, 33 in Group B, and 42 in Group C. The blockages were primarily located at the fimbrial level, affecting 84 patients. In 21 patients, the blockage was in the ampullary region, and in 12 patients, it was in the isthmic area. Furthermore, 54 patients had bilateral tubal defects, with the majority of defects located at the fimbrial level.

The patients were followed up by phone to monitor their fertility outcomes at six and 12 months post-intervention. A total of 63 patients became pregnant. The correlation analysis suggested that tubal defect characteristics and age category significantly influenced fertility outcomes. The best fertility results were observed in age category A. Another negative factor for pregnancy success was the patient's BMI, with a p-value of 0.01 (less than 0.05), Out of the successful fertility outcomes, 52 occurred within six months post-intervention, and 11 occurred within the subsequent 12 months. Among those who became pregnant, 24 experienced secondary infertility. Intraoperative observations revealed various findings among patients who successfully conceived, including 26 with both fallopian tubes patent and 35 with blockage in a single fallopian tube. Only two patients who achieved pregnancy had bilateral tubal blockage; in these cases, a bilateral salpingostomy was performed, allowing the patients to successfully conceive despite the initial bilateral blockage.

**Figure 3:**
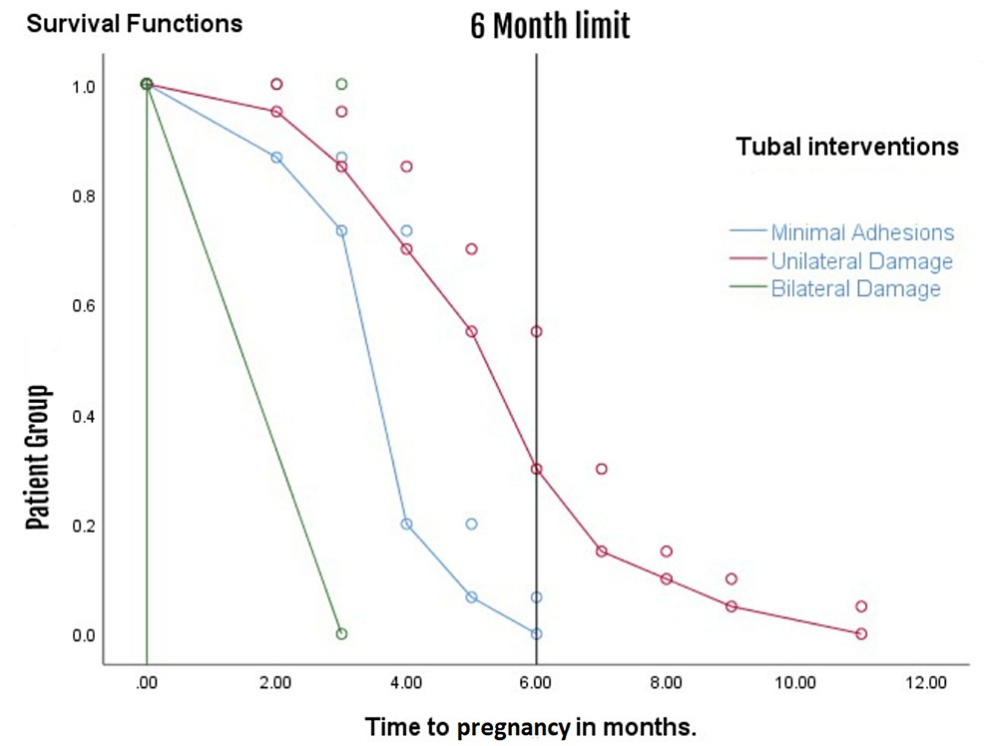
Survival curve showing the patients' chance of getting pregnant depending on the intervention performed within 12 months.

Figure 3 is an illustration representing a span of 12 months, displaying the number of patients who achieved pregnancy categorized by the type of intervention performed, as estimated by the Kaplan-Meier methodology.

The displayed chart clearly shows that most successful pregnancy outcomes took place within the initial six months after the intervention. Additionally, our results suggest that patients who underwent unilateral salpingostomy experienced higher fertility rates compared to others who received different treatments. Unfortunately, we cannot discuss the chances of success in achieving pregnancy in the patients who required anastomosis (n=18), because none of them achieved pregnancy.

## Discussion

Our findings reveal that the most favorable outcomes were observed among patients in the 20-25 age group, which is consistent with existing knowledge in reproductive medicine that highlights age as a significant factor impacting fertility [3,4]. As for the patients' weight, the distribution was fairly equal, with only 12 patients classified as obese. This small sample size makes it difficult to study the effect of this parameter on the results. However, among the obese patients, six were successfully conceived, including four in the 20-25 age group with primary infertility and two in the 30-35 age group with secondary infertility. Being overweight or obese is known to negatively affect fertility [5]. Additionally, the relationship between BMI and age has been shown to have a considerable influence on the outcomes of in vitro fertilization [6].

In our study, the number of patients with primary infertility who managed to get pregnant was higher than those with secondary infertility, but the diagram shows how a positive obstetric history influences the chance of getting pregnant Consequently, younger patients with a positive obstetric history have the highest probability of success, while those with primary infertility in the 30-35 age range have the lowest chance. For patients with pelvic inflammatory disease (PID), the antecedents or asymptomatic infections diagnosed preoperatively could not be shown to have an effect on the fertility outcome in our study, instead, by performing a correlation between the intraoperative observations and PID, it was observed to have a p-value of -0.15, with a strong relationship between the two variables. Multiple studies demonstrate the impact of PID on fertility [7], but the authors believe that the age at which the disease appears plays a role in the impact of the disease on the fallopian tubes [8].

A number of 47 patients were found with intraperitoneal adhesions and with reduced mobility of the fimbrial area which is an often overlooked cause of infertility. Of these, most were in Group A (n=24), the rest in Group B (n=16), and Group C (n=7), of these, 26 patients (55.31%) got pregnant, and it took a very short period of time after the intervention for these patients to conceive (with mean of 3.96 months). The results also showed 117 patients with unilateral tubal defects of which 35 got pregnant, which represents 29.91% of the population with a mean time of 5.75 months until conception; two patients got pregnant out of 54 patients with bilateral tubal blockage, which represents 3.70%. Another unpleasant observation was the fact that all the patients who were successful after salpingostomy had a defect at the fimbrial level in the distal 2 centimeters, and none of those who got pregnant had a more proximal tubal defect. After surgery, the projected live birth rates vary between 9% for women with serious tubal issues and 69% for those with mild conditions [9]. Our results showed a 55.31% chance for those with mild conditions and 3.70% for serious tubal issues. Also, our results from the study show that the chances of conception in young patients (20-25 years) were not significantly influenced by other parameters such as higher BMI (overweight) or sexually transmitted infections, probably due to their reduced influence on fertility at this age; however, in the 30-35 age group we can conclude that the history of sexually transmitted infection had a major impact on tubal permeability, and an important observation was that in the second half of following up (from 6-12 months) the number of patients who conceived was low, which is similar to some observational studies [10,11] and in contradiction with others that are carried out over a longer period [12].

Limitations of the study

Regarding the limitations of the study, one is the small number of the study population; therefore, further studies are recommended to be conducted using a larger sample size in this regard.

## Conclusions

With advancing age, the severity of tubal disorders increases, so age, parity, and the severity of tubal damage are the most important factors predicting the success of tubal intervention. Fimbrolysis demonstrated the highest fertility rate, with a success rate of 55.31% among patients. Salpingotomy was next, showing varied outcomes depending on the need for intervention on one or both fallopian tubes; the conception rate was 29.91% for patients requiring intervention on a single tube and 3.70% for those needing intervention on both tubes. In our group, simple salpingostomy conducted within the following 2 cm of the ampullary region and anastomosis performed in the isthmic area did not result in successful conception. The time to conception shows that the mean time for conception was 4.8 ±2.1 months with a mere 21.15% success rate; these individuals became pregnant within a timeframe of 6-12 months.

The optimal duration to ascertain the possibility of pregnancy following a tubal intervention remains undefined. However, evidence from our study suggests that beyond a 12-month period, the likelihood of conception significantly diminishes post-intervention. Therefore, it is prudent to consider a period of 12 months as the upper limit for anticipating a successful pregnancy post-procedure.
